# Anaphylaxis to beetroot (*Beta vulgaris*): a case report

**DOI:** 10.1186/2045-7022-1-S1-P51

**Published:** 2011-08-12

**Authors:** Lucila Camargo Lopes de Oliveira, Isabel Ruguê Genov, Elza do Carmo Cabral, Yara Arruda MF Mello, Márcia Carvalho Mallozi, Dirceu Solé

**Affiliations:** 1 UNIFESP, São Paulo, Brazil; 2 Clínica de Alergia São Paulo, São Paulo, Brazil

## Background

Allergy to beetroot is very rare. Until now only a few reports about asthma and rhinoconjunctivitis induced by inhaling the vapor of cooked beet have been reported.Oral food challenging on any non-toxic adverse reaction to food may be important.

## Methods

Record review.

## Results

LSJ, a 13-years old Brazilian girl has complained of urticaria and asthma about 40 minutes after ingesting salty boiled beetroot on a meal. She had a previous history of wheezing until she was five years old. Seric-specific IgE to beetroot and other foods were negative, as well as to latex, bee, wasp and ant. She was sensitized only to house dust mite. Boiled and raw beetroot prick to prick were also negative (histamine resulted 4mm) but a food challenge with boiled beetroot resulted positive after 30 minutes with generalized hives, throat tightness and bronchospasm. Symptoms were controlled with intramuscular epinephrine, inhaled beta-2-agonists and intravenous corticosteroids. She was advised for a diet free of beetroot and she has had no symptoms for the last 8 months.

## Conclusions

Although beetroot sensitization was not proven, one must still consider in this case an IgE-mediated reaction against a neoallergen promoted by protein digestion or food intolerance. In both situations a food challenge is necessary. Discharging patients based only on a negative sensitization investigation may be life threatening.

